# Postpartum Exudation of Idiopathic Quiescent Macular Neovascularization: A Narrative Review with a Related Case Report

**DOI:** 10.3390/life15010031

**Published:** 2024-12-30

**Authors:** Livio Vitiello, Maddalena De Bernardo, Ilaria De Pascale, Giulio Salerno, Alfonso Pellegrino, Nicola Rosa

**Affiliations:** 1 Eye Unit, “Luigi Curto” Hospital, Azienda Sanitaria Locale Salerno, 84035 Polla, SA, Italy; idepascale@outlook.it (I.D.P.); giuliosalerno@hotmail.it (G.S.); al.pellegrino@aslsalerno.it (A.P.); 2 Eye Unit, Department of Medicine, Surgery, and Dentistry “Scuola Medica Salernitana”, University of Salerno, 84081 Baronissi, SA, Italy; mdebernardo@unisa.it (M.D.B.); nrosa@unisa.it (N.R.)

**Keywords:** idiopathic macular neovascularization, MNV, oxytocin, pregnancy, quiescent macular neovascularization

## Abstract

The abnormal growth of irregular new blood vessels into the subretinal or intraretinal space is known as macular neovascularization (MNV). People over 50 are often affected by this disorder, which is typically brought on by age-related macular degeneration. In addition, MNV can be found in people under 50 years of age, who may present primary ophthalmic diseases such as pathological myopia, angioid streaks, traumatic choroidal rupture, or suspected ocular histoplasmosis syndrome. However, it is important to consider a specific set of young individuals who may develop MNV even in the absence of pathological myopia or other identifiable inflammatory, peripapillary, post-traumatic, or degenerative fundus abnormalities. This latter condition is classified as idiopathic MNV. After a literature review focused on young patients affected by one of these two clinical entities, we report the case of a Caucasian young woman suffering for four years from an idiopathic and quiescent MNV that started exuding after childbirth, probably due to the induction with oxytocin, and was treated with intravitreal Aflibercept 2 mg injections.

## 1. Introduction

Macular neovascularization (MNV) indicates the pathological development of irregular new blood vessels into the subretinal or intraretinal space. This retinal disease usually affects people over 50 years old and is generally caused by age-related macular de-generation (AMD) [1]. In younger patients, MNV is usually associated with primary ophthalmic disorders such as pathological myopia, angioid streaks, traumatic choroidal rupture, or presumed ocular histoplasmosis syndrome (POHS) [1,2].

However, a distinct group of young patients, in whom MNV develops without pathological myopia or recognizable inflammatory, peripapillary, post-traumatic, or degenerative fundus findings, should be considered. This condition is recognized as idiopathic MNV [3,4,5,6].

Among the retinal diseases, quiescent MNV has been described as an angiographically detected MNV, with both fluorescein angiography (FA) or indocyanine green angiography (ICGA), showing absence of intraretinal/subretinal exudation during repeated optical coherence tomography (OCT) follow-up examinations (at least two evaluations, with a six-month interval) [7]. On FA, quiescent MNVs appear as late-sprinkled hyperfluorescent lesions lacking well-demarked boundaries, without late-phase leakage of undetermined source or dye pooling in the subretinal space. On the other hand, mid-late phase ICGA frames allow visualization of the hyperfluorescent quiescent MNV network and delineation of the plaque [7].

Concerning OCT, quiescent MNVs present as an irregularly slightly elevated retinal pigment epithelium (RPE), without hyporeflective fluid accumulation in the intraretinal/subretinal area, showing a large axis in the horizontal plane, and distinguished by collections of moderately reflective material in the sub-RPE area and strong visualization of the Bruch hyperreflective membrane [7]. Furthermore, in quiescent MNV no other signs of activity are detectable, such as mid-reflective exudative content, low optical lesion reflectivity/poorly defined lesion boundaries, and intraretinal hyperreflective flecks [8]. Asymptomatic treatment-naïve quiescent MNV may be generally untreated until intraretinal/subretinal exudation on OCT develops [7].

In the current literature, different case reports on idiopathic exudative MNV in young patients have been reported but, to the best of our knowledge, no cases of idiopathic quiescent MNV in young women that started exuding after the childbirth have been discussed.

For this reason, the main purpose of this paper is to briefly summarize these two different clinical entities, idiopathic and quiescent MNV, illustrating their clinical and diagnostic features through a literature review focused on young patients affected by one of these clinical entities; then, we present a unique case of a young woman with a coexisting idiopathic and quiescent MNV that started exuding after childbirth with oxytocin induction and was treated with intravitreal Aflibercept 2 mg injections; finally, we examine the scientific literature to try to find evidence of a correlation between hormones and retinal diseases and, in particular, between oxytocin and MNVs.

## 2. Idiopathic MNV

### 2.1. Definition

Idiopathic MNV is generally a monocular condition which primarily affects individuals under the age of 50 [9]. The primary cause of visual impairment is the development of MNV in the macular region, followed by exudation, hemorrhage, and scarring. This is similar to the wet AMD pathophysiology, but without any definite cause, such as pathological myopia, POHS, angioid streaks, or traumatic choroidal rupture [1,2,10]. According to one previous study, people affected by idiopathic MNV had considerably greater serum vascular endothelial growth factor (VEGF) concentrations than a control group [10].

The etiology of idiopathic MNV cannot be used to treat the disorder since its pathophysiology is yet unknown [11,12].

### 2.2. Diagnosis and Treatment

Concerning the symptoms, they are similar to those related to other MNV forms: sudden decrease in visual acuity, with associated distortion and blurring of central vision, metamorphopsia, micropsia, photopsia, decreased color vision, and xanthochromia [9]. Regarding the diagnosis, as in all other cases of MNVs, idiopathic MNV is likewise identified with multimodal imaging, especially with the OCT examination, in addition to fundoscopic evaluation, FA, and ICGA [2].

Anti-VEGF treatment is currently used to treat idiopathic MNV similarly to wet AMD. In particular, anti-VEGF intravitreal injections (once a month for three months) on a “treat and extend” regimen constitute the conventional treatment for wet AMD [13]. Conversely, idiopathic MNV usually presents smaller MNV lesions than in AMD, with fewer hemorrhages and less edema. For this reason, the 1 + pro re nata treatment regimen is typically used to treat patients with idiopathic MNV [14].

### 2.3. Idiopathic MNV in Young Patients

In the published scientific literature, the presence of idiopathic MNV in young patients has been discussed in several case reports.

Mohammadpour et al. [15] described the case of a 23-year-old girl with blurred vision and metamorphopsia in her left eye in the past 2 months. Her past medical history was unremarkable. On presentation, her left-eye best corrected visual acuity was 20/80, with a normal anterior segment examination. Posterior segment evaluation showed an ill-defined green-gray elevation in the fovea with surrounding subretinal fluid. FA and OCT were performed, confirming the presence of an exudative idiopathic MNV, subsequently treated with only one intravitreal bevacizumab injection, with a complete resolution of the exudation one month after the intravitreal therapy.

Khan and colleagues [16] discussed the case of a 36-year-old male patient with blurred vision and metamorphopsia in his left eye for six weeks. His past medical, systemic and ocular history was unremarkable. On examination, his best corrected visual acuity was 20/20 and 20/60 in his right eye and left eye, respectively, with a normal anterior segment evaluation in both eyes. Fundus examination of the left eye showed a juxtafoveal inferotemporal greyish-white elevated lesion approximately 1/3rd disc diameter, with specks of subretinal hemorrhages bordering the lesion. FA showed the lesion as a well-defined lacy pattern of hyperfluorescence in the early phase with increasing leakage in the late phase, while OCT showed a subretinal hyperreflective membrane with a few small intraretinal cystic areas. He was diagnosed with idiopathic MNV and treated with three intravitreal ranibizumab injections, with a full recovery and a visual acuity of 20/20 at the final follow-up visit (six months after the last injection).

Waheeb and Showail [17] described the case of a 17-year-old female patient with no significant past medical history, who anyway presented an idiopathic exudative MNV. On the initial examination, she could only count fingers at one meter in the left eye, and FA showed a well-defined hyperfluorescent area corresponding to the neovascular membrane. Intravitreal bevacizumab was injected and, at a five-week follow-up visit, visual acuity improved to 20/100.

### 2.4. Idiopathic MNV in Pregnant Young Women

Anastasilakis et al. [18] presented the case of a 31-year-old woman in the eighth month of her second pregnancy, with mild macular and papillary edema. She was followed up utilizing biomicroscopy, FA, and OCT, showing a worsening three months after childbirth with the appearance of a juxtapapillary MNV. After two intravitreal ranibizumab injections, her best corrected visual acuity increased significantly, physiological macular anatomy was restored, and no subretinal fluid was observed.

Monis and colleagues [19] reported the case of a 27-year-old pregnant woman who had complained of diminished and blurry vision in her left eye for the previous two weeks, with a visual acuity of 20/63, and no further improvement. The diagnosis of idiopathic MNV in pregnancy was made after carrying out a thorough ophthalmological examination, clinical history, and investigations. Despite receiving a lot of counseling, the patient refused the anti-VEGF therapy because of potential harmful consequences for the fetus. For this reason, she was told to get intravitreal anti-VEGF injections as soon as she delivered, but she was lost at follow-up.

Likewise, Fossum et al. in their case series [20] described an exudative recurrence of idiopathic MNV in a 26-year-old woman with a medical history of miscarriages, who complained of blurred vision and metamorphopsia. Her OCT showed macular edema with serous macular neurosensory detachment, and her best corrected visual acuity was 20/40. With the patient’s agreement, an intravitreal ranibizumab injection was given at 10 weeks after the last menstrual period and again at 21 weeks after the last menstrual period, with the resolution of the macular edema, and without adverse events.

De Silva and co-authors [21] presented a case report of a 29-year-old pregnant woman who, at 26 weeks along, experienced distortion and blurry vision in her left eye, with a best corrected visual acuity of 20/125. She was diagnosed with idiopathic MNV, and several potential therapy options were reviewed with the patient. However, the patient was unwilling to take any chances by using an anti-VEGF drug while she was pregnant, so she decided to have an early birth at 32 weeks after consulting with the obstetric team. Then, she underwent an intravitreal bevacizumab treatment, with a complete resolution of the visual symptoms and the intraretinal and subretinal fluid.

Jouve et al. [22] reported the case of a 29-year-old woman who noted severe blurriness in her left eye during her seventh month of pregnancy, resulting in a decrease in visual acuity from 10/20 to 6/20 in just one month. Fundoscopy revealed intraretinal white zones next to the optic disc, while OCT showed a serous neurosensory detachment involving the fovea. Considering the juxtapapillary idiopathic MNV shown on the FA, the authors chose to provide a single intravitreal ranibizumab injection to the woman during her pregnancy. Due to persistent peripapillary subretinal fluid following an uncomplicated labor, the patient had two further intravitreal anti-VEGF injections while breastfeeding was halted. The best corrected visual acuity was steady at 20/20 six months following the last injection, with just a little suprapapillary fibrotic scar and no residual subretinal fluid.

Finally, Sarhianaki and colleagues [23] diagnosed a subfoveal idiopathic MNV in the right eye of a 29-year-old pregnant woman. Her best corrected visual acuity was 20/80, with the presence of intraretinal edema and a round grey-yellowish lesion associated with subretinal hemorrhage in the center of the macula. For this reason, two intravitreal ranibizumab injections were carried out after monthly OCT evaluation, with best corrected visual acuity improving to 20/25 and with a complete regression of the fluid, except for a small residual macular scar.

## 3. Quiescent MNV

### 3.1. Definition

In 2013, Querques et al. [7] defined as “quiescent” those subclinical, treatment-naïve, type 1 macular neovascularization lesions in the absence of frank exudation on OCT for at least 6 months [7]. The Latin word “quiescentia”, which means “inactivity, without symptoms”, is whence the word “quiescent” originates [7,24,25]. The 6-month follow-up period was selected at random as it corresponds to the clinical follow-up of patients with significant drusen (intermediate AMD). At that time, patients with quiescent MNV were then classified as intermediate AMD since there was no exudation [25].

Subsequently, the CONAN group promoted the use of the term non-exudative MNV, as no agreement on the word “quiescent” was reached [26]. Beyond nomenclature, a type 1 MNV without intraretinal or subretinal exudation on repeated OCT B-scans for at least 6 months is referred to as non-exudative or “quiescent” MNV [7,24].

Non-exudative neovascularization lesions are typically found in the setting of intermediate AMD, but they can also be linked to other abnormalities in the macular structure, such as angioid streaks, geographic atrophy, pachychoroid neovasculopathy, and large colloid drusen [27,28,29,30].

### 3.2. Diagnosis of Quiescent MNV

At dye angiography, quiescent MNVs are characterized by “late speckled hyperfluorescent lesions lacking well-demarcated borders (without pooling of dye in the subretinal space or late-phase leakage of an unknown source, which defines typical occult type 1 MNVs)” [7]. Additionally, in the mid-late phase of ICGA, quiescent MNVs show up as a distinct plaque, similarly to typical exudative type 1 MNVs [7].

In structural OCT, quiescent MNV is characterized by the visualization of two hyperreflective layers (RPE and the Bruch membrane) with the major axis on the horizontal plane, and with the presence of reflective material inside, in the absence of subretinal fluid. This is known as “double-layer sign” [31,32]. Furthermore, Narita et al. [33] further expanded the features of the double-layer sign characterizing non-exudative MNVs and introduced the term shallow, irregular RPE elevation (SIRE). SIRE is characterized by a double layer sign with non-homogeneous internal reflectivity and by RPE elevation greater than 1000 μm in horizontal length and less than 100 μm in height [33].

Conversely, considering OCT angiography (OCTA), several limits, like projection artifacts and the OCTA difficulties in the detection of flow that is either too fast or too slow, must be considered in cases of quiescent MNV evaluation. In fact, in an avascular pigment epithelial detachment (PED), projection artifacts may lead to the incorrect identification of a neovascular network. On the other hand, OCTA could miss non-exudative neovascularization with a very sluggish flow [34]. OCTA can distinguish between a drusenoid PED, which arises from the convergence of soft drusen, and a fibrovascular PED [34]. Lesion types with irregular shapes, non-visible cores, well-defined edges, and foveal sparing were the most common ones seen on OCTA [25]. As previously recommended, the lack of exudative traits for a minimum six-month interval was deemed to be considered as non-exudative neovascularization [7,35].

Currently, no clinical cases of previously diagnosed quiescent MNVs that started exuding during or immediately after pregnancy are present in the published scientific literature.

## 4. Case Description

A 34-year-old Italian woman came to our Eye Unit for an annual routine comprehensive ophthalmological evaluation in September 2019. Her ocular history revealed previous strabismus surgery in both eyes when she was three years old. She was healthy, with no history of systemic disease, drug use, or ocular trauma, and a previous uneventful pregnancy three years earlier.

Her best corrected visual acuity was 20/20 in both eyes (right eye: sph +2.5 cyl +3.5 α 100; left eye: sph +1.75 cyl +3 α 80), the anterior segment evaluation was unremarkable in both eyes, and the intraocular pressure was 12 mmHg in her right eye and 10 mmHg in her left eye. The axial length of both eyes was additionally measured with an IOLMaster 700 (Carl Zeiss Meditec AG, Jena, Germany) to further exclude axial myopia, indeed showing 20.68 mm in the right eye and 20.74 mm in the left eye.

**Figure 1:**
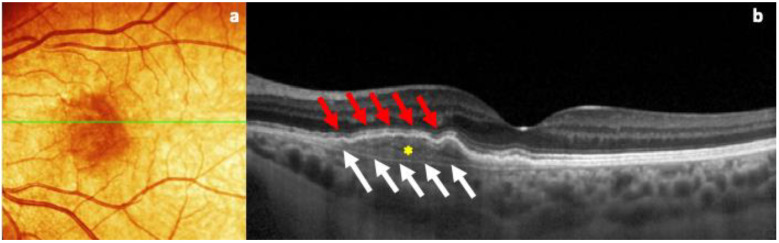
(**a**) OCT color fundus image of the quiescent MNV, showing the presence of slightly hyperpigmented flat lesion, with indefinite edges, in the macular region. (**b**) B-scan OCT image of the same retinal lesion, characterized by the visualization of two hyperreflective layers (double-layer sign): retinal pigment epithelium (red arrows) and the Bruch membrane (white arrows) with a major axis on the horizontal plane, with the presence of reflective material inside (yellow asterisk), in the absence of subretinal fluid.

In the fundus examination, the right eye was healthy, with regular tortuosity of the retinal vessels, a trophic macula, and a normal optic disc, in the absence of retinal lesions. On the other hand, the left eye showed an RPE alteration with a slight yellowish retinal elevation in the interpapillo-macular region. For this reason, an OCT evaluation (Spectralis; Heidelberg Engineering; Heidelberg, Germany, version 6.0) was performed to better determine this retinal finding, which revealed an irregularly slightly elevated RPE, characterized by collections of moderately reflective material in the sub-RPE area and strong visualization of the Bruch hyperreflective membrane with a large axis in the horizontal plane, with no signs of exudation (Figure 1).

The patient was followed up monthly with visual acuity evaluations and fundus examinations to identify any potential changes in clinical characteristics until February 2020 when, due to the COVID-19 pandemic, she was lost to follow-up.

**Figure 2:**
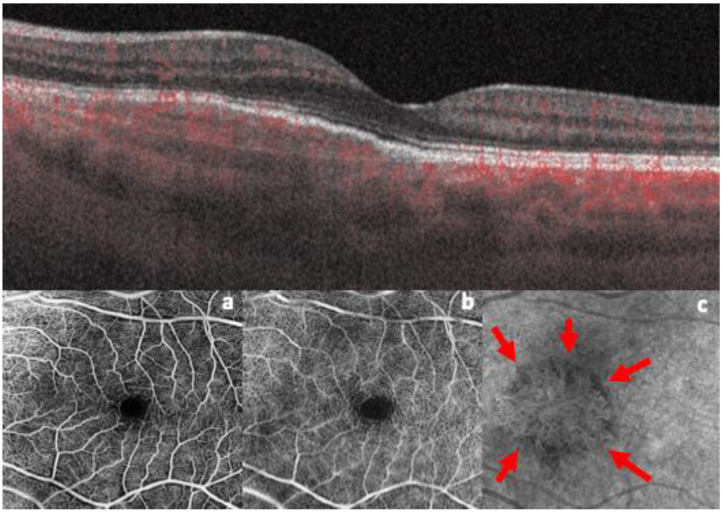
Top image: B-scan OCT of the quiescent MNV, showing the “double-layer sign” and the presence of hyperreflective material under the retinal pigment epithelium, in the absence of subretinal fluid. Bottom images: 3 × 3 mm OCT angiogram of the superficial (**a**) and deep (**b**) capillary plexus. The OCT angiogram of the choriocapillaris (**c**) displays a well-defined neovascular network under the retinal pigment epithelium (red arrows).

In September 2022, though asymptomatic, she underwent a new ophthalmological examination, including an OCTA evaluation (Zeiss Plexelite, Plexelite, Carl Zeiss, Meditec AG, Jena, Germany), which confirmed the diagnosis of an idiopathic quiescent MNV, showing a well-defined neovascular network at the choriocapillaris level in the absence of any exudation (Figure 2).

**Figure 3:**
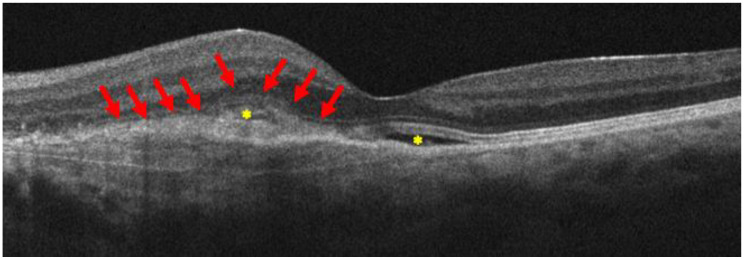
B-scan OCT reveals an enlargement of the known retinal lesion, with a maximum retinal thickness of 433 microns, fuzzy borders, and subretinal hyperreflective material, which is a sign of progression of type 1 to type 2 MNV (red arrows), in the presence of intraretinal and subretinal fluid (yellow asterisks). All are imaging signs of MNV activation.

Three months later, she became pregnant and, in September 2023, eight days postpartum, having been induced with low-dose oxytocin (0.5 to 2 mU/min with incremental increases of 1 to 2 mU/min every 15 to 40 min) [36], she complained of blurred vision and metamorphopsia in her left eye. Her best corrected visual acuity was 20/200, and the OCT revealed the presence of hyporeflective intraretinal and subretinal fluid accumulation nearby the known retinal lesion (Figure 3).

Considering her ocular condition and breastfeeding status, we first asked for National Poison Control Center approval for intravitreal treatment, which specifically recommended the use of aflibercept 2 mg. For this reason, the patient was suggested to start anti-VEGF intravitreal injections treatment with aflibercept 2 mg, until retinal fluid resolution was achieved, for a total of five monthly intravitreal injections.

Two months after the fifth intravitreal injection, the visual acuity in her left eye improved to 20/20, even if she still complained of metamorphopsia. An OCT examination revealed an improvement compared to the previous control, but small intraretinal cysts were still detected near the retinal lesion, which still appeared with fuzzy borders. Therefore, an additional intravitreal injection with aflibercept 2 mg was performed.

One month after the last intravitreal injection, the patient reported a slight improvement in metamorphopsia, even if it was still present, and her visual acuity was stable at 20/20.

**Figure 4:**
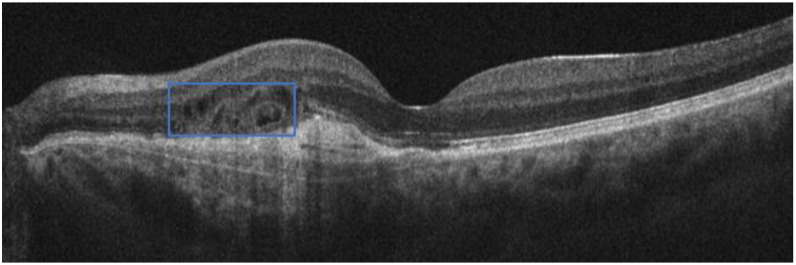
OCT B-scan one month after the sixth intravitreal injection. The maximum retinal thickness decreased to 337 microns, and it is possible to detect the presence of some outer retinal tubulations and small intraretinal cysts (blue square), with more regular borders of the retinal lesion and a decrease in the fibrous scar area.

The OCT evaluation similarly showed a slight improvement in intraretinal cysts, detecting more regular borders of the retinal lesion and a decrease in the fibrous scar area, together with the presence of some outer retinal tubulations, indicating a rearrangement of photoreceptors, as a consequence of retinal injury [37] (Figure 4).

For this reason, the patient was suggested to perform a strict month-by-month follow-up to adequately decide if additional treatment was required, following an “observe and extend” protocol [38,39].

Two months after the sixth intravitreal injection, the visual acuity remained stable, but the intraretinal fluid persisted and the patient still complained of mild worsening of metamorphopsia. For this reason, another intravitreal aflibercept 2 mg was administered.

**Figure 5:**
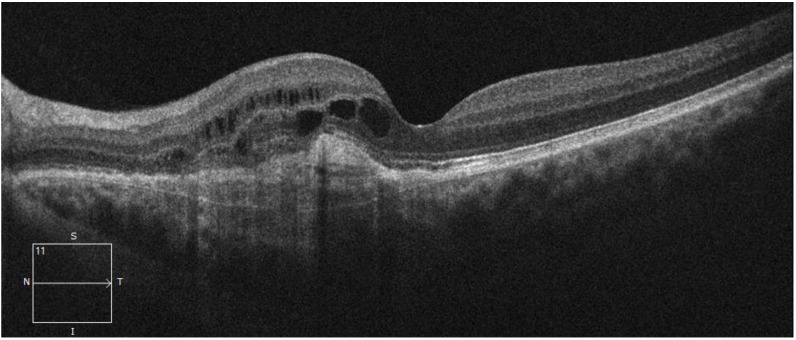
OCT B-scan three months after the last intravitreal injection, showing a retinal thickness increase (368 microns) and a recurrent worsening of intraretinal fluid, associated with a concomitant worsening of visual acuity and metamorphopsia.

The therapeutic effect of the last intravitreal injection lasted for almost 3 months, when a further worsening of visual acuity to 20/30, intraretinal fluid (Figure 5), and metamorphopsia were found.

For this reason, an eighth intravitreal injection was necessary, with the patient still currently being monitored monthly.

## 5. Discussion

Nowadays, idiopathic MNV is a well-defined clinical entity characterized by its presence in young patients (under 50) with sudden onset of symptoms related to the submacular neovascular membrane, such as metamorphopsia and decreased visual acuity, and with no identifiable primary or systemic disease (Table 1).

In this case report, we have discussed the presence of an idiopathic quiescent MNV in a young woman who began exuding after partum with oxytocin induction. To date, no similar case reports have been discussed in the published scientific literature, while several papers have reported on idiopathic and quiescent MNV separately in young patients and during pregnancy [7,15,16,17,18,19,20,21,22,23,28,40,41].

In the present case report, the young patient met all the conditions underlying an idiopathic MNV but, differently from the above-mentioned reports [15,16,17,18,19,20,21,22,23], without signs of activity and without any clinical signs for almost 4 years, including the second pregnancy period. In fact, her neovascular membrane had all the OCT signs of a typical quiescent form: an irregularly slightly elevated RPE, without hyporeflective fluid accumulation in the intraretinal/subretinal area; a large axis of the neovascular membrane in the horizontal plane, characterized by collections of moderately reflective material in the sub-RPE area; and a strong visualization of the Bruch hyperreflective membrane [7].

Moreover, no changes had been detected in the features and in the activity of this neovascular membrane for more than 6 months, thus satisfying another important criterion for the definition of quiescent or non-exudative MNV [7].

Furthermore, even though FA and/or ICGA were not performed, OCT and especially OCTA played a key role in the initial characterization of the quiescent lesion of this patient. In fact, several studies have demonstrated a good correspondence of the sensibility and specificity of OCTA in the detection of quiescent MNV when compared with traditional dye angiography [28,40,41].

Another peculiarity of this case is the possible association of macular exudation immediately after childbirth with oxytocin induction. The association between hormones and ocular diseases, especially the retinal ones, have been already described in the scientific literature. In particular, mineralocorticoids and melatonin seem to be involved in the pathogenesis, management, and therapy of some retinal pathologies, including central serous chorioretinopathy and other diseases belonging to the pachychoroid spectrum [42,43,44,45,46,47]. Furthermore, sex hormone deficiency has been demonstrated to be related to an increase risk to develop glaucoma and AMD, while progesterone and estradiol have been shown to be effective in protecting photoreceptors [48]. However, the underlying mechanisms are not yet known and are currently being investigated.

Moreover, pregnancy is also considered a risk factor for valsalva retinopathy, which typically presents as a sudden visual loss caused by a premacular hemorrhage [49]. Nonetheless, our patient did not have a premacular hemorrhage, but a perilesional exudative process of the previously diagnosed retinal lesion.

Pregnancy is characterized by continuous hormonal imbalances, which can have several effects on different organs, including the eye [50]. In our opinion, the exudation of the quiescent MNV in this young lady may be linked to hormonal changes immediately following the end of pregnancy, along with the oxytocin utilized for induction, as the presence of an oxytocin signaling pathway at the RPE level is already known [51,52]. In fact, oxytocin has been demonstrated to serve as a paracrine signaling pathway that may contribute to communication between the cone photoreceptors and the RPE, also involving dopaminergic pathway [51,53]. For this reason, it cannot be excluded that imbalances in the body’s levels of oxytocin could lead to an alteration of ocular homeostasis, especially in the presence of a previous MNV. Nevertheless, this hypothesis remains only speculative, since there are currently no research studies in the scientific literature on the possible correlation between oxytocin levels and MNVs.

In addition, the hypothesis of oxytocin involvement, as well as the persistence of intraretinal fluid despite several intravitreal aflibercept 2 mg injections, could be further supported by the special condition of the patient, i.e., that she was still breastfeeding. Indeed, newborn sucking is known to be involved in oxytocin production [54].

However, very little is known about the effects of oxytocin and other hormones on the retinal structures and, in particular, their involvement in the pathogenesis of MNVs. Therefore, further studies are needed to more deeply investigate these potential implications.

In future research, given the inefficacy of intravitreal aflibercept 2 mg in this patient, it could be evaluated whether there is an improvement in intraretinal fluid and metamorphopsia after the cessation of breastfeeding, with a concomitant reduction in oxytocin levels. In addition, at the end of breastfeeding, a switch to new intravitreal anti-VEGF drugs, such as faricimab and aflibercept 8 mg, could be also considered.

Finally, the main limitations of this report are its single-case design and the speculative nature of oxytocin’s association with MNVs.

## 6. Conclusions

In conclusion, this case report suggests the possibility to detect an idiopathic quiescent MNV in young patients, where a strict follow-up is strongly recommended to early identify the possible progression of the lesion. Furthermore, an “observe and extend” protocol could represent the best tailored choice in case of pregnant women or breastfeeding, also considering that anti-VEGF treatment during pregnancy is associated with child loss and therefore contraindicated.

Finally, intravitreal treatment with aflibercept 2 mg appeared to be harmless both for the young breastfeeding patient and for the newborn, since no adverse events were detected throughout the therapeutic period. However, additional clinical studies are needed both to demonstrate the safety of anti-VEGF drugs in these conditions and to further understand the possible role of hormones in the onset of exudation of previously quiescent MNVs.

## Figures and Tables

**Table 1:** Summary of the main clinical and diagnostic features of idiopathic MNV and quiescent MNV.

	Idiopathic MNV	Quiescent MNV
**Onset**	Under 50 years	Over 50 years
**Symptoms**	Decreased visual acuity Metamorphopsia Micropsia Photopsia Decreased color vision	Typically asymptomatic
**OCT/OCTA**	Presence of different signs of exudation, depending on type of MNV	Presence of two hyperreflective layers (RPE and the Bruch membrane) with a major axis on the horizontal plane, and with the presence of reflective material inside, in the absence of subretinal fluid (double-layer sign). Presence of a well-defined neovascular network, in the absence of exudation
**FA**	Presence of dye pooling, depending on type of MNV	Late speckled hyperfluorescent lesions lacking well-demarcated borders, without pooling of dye in the subretinal space or late-phase leakage of an unknown source
**ICGA**	Presence of dye pooling, depending on type of MNV	In the mid-late phase, the lesion appears as a distinct plaque, similarly to typical exudative type 1 MNV
**Treatment**	Intravitreal anti-VEGF drugs	No therapy

MNV: Macular neovascularization; OCT: Optical coherence tomography; OCTA: Optical coherence tomography angiography; FA: Fluorescein angiography; ICGA: Indocyanine green angiography; VEGF: Vascular endothelial growth factor.

## Data Availability

No new data were created or analyzed in this study. All the data are contained within the article.
